# Lightweight Meter Pointer Recognition Method Based on Improved YOLOv5

**DOI:** 10.3390/s24051507

**Published:** 2024-02-26

**Authors:** Chi Zhang, Kai Wang, Jie Zhang, Fan Zhou, Le Zou

**Affiliations:** 1 School of Artificial Intelligence and Big Data, Hefei University, Hefei 230601, China; hfuu_zc@hfuu.edu.cn (C.Z.);; 2 Institute of Intelligent Machinery, Hefei Institute of Physical Sciences, Chinese Academy of Sciences, Hefei 230031, China

**Keywords:** deep learning, meter reading, object detection, substation patrol

## Abstract

In substation lightning rod meter reading data taking, the classical object detection model is not suitable for deployment in substation monitoring hardware devices due to its large size, large number of parameters, and slow detection speed, while is difficult to balance detection accuracy and real-time requirements with the existing lightweight object detection model. To address this problem, this paper constructs a lightweight object detection algorithm, YOLOv5-Meter Reading Lighting (YOLOv5-MRL), based on the improved YOLOv5 model’s speed while maintaining accuracy. Then, the YOLOv5s are pruned based on the convolutional kernel channel soft pruning algorithm, which greatly reduces the number of parameters in the YOLOv5-MRL model while maintaining a certain accuracy loss. Finally, in order to facilitate the dial reading, the dial external circle fitting method is proposed to calculate the dial reading using the circular angle algorithm. The experimental results on the self-built dataset show that the YOLOv5-MRL object detection model achieves a mean average precision of 96.9%, a detection speed of 5 ms/frame, and a model weight size of 5.5 MB, making it better than other advanced dial reading models.

## 1. Introduction

The automatic reading of power system meters is mainly focused on the intelligent inspection of substation robots. Due to its high accuracy, fast operation, and stability, computer vision technology is now widely used in industrial instrumentation measurement for automatic inspection. This technology eliminates the need for workers to perform complex and monotonous tasks, thus increasing productivity. Typically, a gauge inspection system consists of two stages: Firstly, the region containing the pointer is isolated from the captured image of the instrument panel, and its position and orientation are identified concurrently. Subsequently, the indicator marks are extracted and utilized as a reference for the pointer’s position. The detection system computes the deviation between the provided value and the pointer reading in order to assess the accuracy of the gauge board’s pointer. Finally, meters that do not meet the accuracy criteria will be calibrated. In an earlier study, Alegria et al. [1] performed a subtraction operation on two images, followed by a binarization and refinement operation, and then fitted the pointer line using the Hough transform. Belan et al. [2] introduced a segmentation-free approach that employed radial projection and the Bresenham algorithm to ascertain the pointer’s position in a simulated instrument. However, these methods do not account for the overlap between the pointer and the numbers on the instrument panel. Consequently, the refinement results encompass the contours of the pointer and the digits, resulting in a deviation of the fitted line from the original pointer position. Thus, these methods do not ensure precise fitting outcomes. To determine the position and rotational angle of tick marks, Chi et al. [3] employed the region-growing method to identify the dial region and its center. This algorithm improves the accuracy of the reading results to a certain extent, but it is only applicable to certain instruments where the scale is distributed in the grayscale region. In recent years, Zheng et al. [4] used traditional computer vision methods to read pointer meters in power systems and proposed a new method for automatic meter reading that uses a combination of multiscale Retinex with color recovery at different luminance levels, perspective transformation to obtain a front view of an image taken at any camera angle, and Hough transformation to determine the position of the pointer. The method was able to automatically read the pointer readings of the analog measurement instrument at different luminance levels and camera angles, and achieved good robustness in the test results. Ma et al. [5] employed a conventional machine learning approach to analyze pointer meters. The algorithm exhibits strong adaptability to challenges such as background interference and pointer shadowing. It is employed in the intelligent inspection of substation inspection robots. Krizhevsky et al. [6] proposed the ImageNet dataset, which greatly contributed to the development of artificial intelligence. Additionally, Chi et al. [6] presented a machine-vision-based automatic meter reading method. This method utilizes a region growing algorithm to identify the dial circle center, extracts the pointer through contour fitting, and accurately reads meters with uniform or uneven-scale line distribution.

In recent years, due to the rapid advancement of artificial intelligence technology, deep learning algorithms have made significant breakthroughs and are used to solve challenging problems in several fields. Liu et al. [7] used the Faster R-CNN detection algorithm to locate the meter location for the first time, obtained high-quality images using the feature correspondence algorithm and perspective transformation technique, and detected the pointer position via Hough transformation to achieve a meter reading. Zhang et al. [8] proposed a water meter pointer reading recognition method based on an improved YOLOv4-Tiny network. The method has strong robustness and high accuracy, and can be easily deployed on mobile devices, but the inference time of 370 ms does not meet the real-time requirement. Wang et al. [9] proposed a deep-learning-based pin counter recognition method to resolve the difficulty of recognition under different complex lighting conditions. The method can effectively identify meter readings across varying lighting conditions and camera angles in the inspection video, offering an advanced meter recognition solution for UAV inspections. Hou et al. [10] introduced an innovative meter reading recognition approach utilizing wireless sensor networks (WSNs) and lightweight convolutional neural networks (CNNs). The technique conducts image preprocessing, CNN classification, and reading computation at the WSN end node. It then transmits only the recognition results to the WSN to minimize the data transmission load. Gao et al. [11] presented a resilient character segmentation classifier comprising a HOG/SVM binary classifier, a character filter, and a HOG/multi-class SVM numeric classifier cascade tailored for recognizing digital characters on a dashboard. Additionally, the scale lines are extracted according to the recognition results. Finally, a Newton interpolation linear relationship is established to diagnose any potential response errors of the meter. Zuo et al. [12] segmented Mask-RCNN for the pointer and automatic reading recognition of pointer meters, and the method enables high-accuracy meter recognition.

Most of the above meter reading studies are based on the improvement of the classical deep learning model, which has the characteristics of deep layers, large size, and many parameters, limiting its application in mobile devices like substation robots, especially in tiny monitoring devices, where oversized models and redundant parameters not only cause a waste of storage resources, but also have a negative impact on operation speed and endurance time. Currently, lightweight models have been proposed, such as Ting-YOLO [13], YOLO-Nano [14], GhostNet [15], etc., but they are mostly used in smart agriculture [16,17] and the smart city field [18,19], and less in meter identification.

In order to solve the problems of current deep-learning-based meter reading models with large size, many parameters, and difficulty in meeting real-time requirements on mobile devices, this paper proposes an improved YOLOv5 object detection model, YOLOv5-MRL, and uses it for lightning rod meter reading in power systems. In this paper, we first replace CSPDarknet convolution with Ghost convolution to achieve the initial compression of the network. Then, the YOLOv5 network is pruned via a soft pruning algorithm of convolutional kernels to remove the convolutional kernels that are not important for meter detection. Further compression of the network is achieved, compressing the size of the model with little loss of model accuracy. The final network prediction output is performed using the model to obtain the class of the target as well as the location coordinate information, and the above information is fed into the meter reading algorithm to the final meter scale. This lightweight model not only improves detection speed and practicality, but also enables the accurate implementation of the meter reading task. In this paper, the YOLOv5-MRL is evaluated on the Lighting-Meter lightning rod dataset for size, number of parameters, and detection speed, and finally verified to meet the accuracy and real-time requirements of meter reading.

## 2. Related Work

### 2.1. Object Detection

Object detection is an important problem in the field of computer vision, which involves the task of automatically identifying and localizing objects in images or videos [20]. In the last few years, deep learning methods have become mainstream in the field of object detection, which have led to significant improvements in the performance of object detection algorithms. The main idea of deep learning methods in object detection is to automate object detection by feeding images into neural networks for convolutional feature extraction and operations such as classification and regression. Currently, deep learning-based object detection algorithms can be divided into two main categories: two-stage detection-based methods and single-stage detection methods. Region-based methods (RBMs) extract candidate regions in an image and then perform classification and regression on these regions. Algorithms such as the RCNN series, Fast R-CNN [20], and Faster R-CNN [21] are typical region extraction-based methods. These algorithms perform convolutional feature extraction and classification for each candidate frame by dividing the image into several candidate frames (region proposal), followed by regression. The advantage of these algorithms is their high accuracy and ability to detect very small targets, but the disadvantage is their slow speed. Single-shot detection methods (SSDMs) entail the direct prediction of a target location and class in an image. Algorithms such as YOLO series, SSD [22], and RetinaNet [23] are typical single-stage detection methods. These algorithms input the image into a neural network and perform both classification and regression operations, allowing them to achieve real-time object detection. These algorithms have the advantage of being fast, but sometimes have low accuracy in detecting small targets or targets with complex shapes. In June 2020, the Ultralytics team introduced the YOLOv5 [24] model, which contains four versions of different sizes: YOLOv5l, YOLOv5s, YOLOv5x, and YOLOv5m. These different versions of the YOLOv5 model have a similar structure and control the number of network layers and each layer through only two parameters, depth and breadth. The YOLOv5 network consists of the input side, backbone, Neck, and prediction. The backbone network extracts features, while the Neck network integrates these features, and the prediction phase performs regression on the input features to generate prediction results. Although YOLOv5 does not propose a novel model system, it integrates many optimization strategies and training techniques that can continuously improve the detection capability of the YOLOv5 object detection algorithm. In May 2022, the Meituan Visual Intelligence R&D team proposed the YOLOv6 [25] model, which mainly improves backbone, neck, head, and training strategies. Additionally, YOLOv6 optimizes a simplified and more efficient “Efficient Decoupled Head” to minimize extra delay overhead without compromising detection accuracy. Regarding the training strategy, YOLOv6 embraces the anchor-free approach, and integrates the SimOTA label assignment strategy and SIoU bounding box regression loss to enhance detection accuracy. These enhancements empower the YOLOv6 model to deliver exceptional performance in both detection accuracy and speed, holding significant promise for research and industrial applications. The YOLOv7 [26] model was proposed by the author team of YOLOv4 in July 2022, and is an object detection model. The core technologies of the model include a new backbone network, an improved feature pyramid, a more powerful object detection head, and data enhancement and training strategies. The new backbone network adopts classical networks such as ResNet and EfficientNet, and makes full use of techniques such as residual connectivity and deep separable convolution to effectively reduce model parameters and computation. The improved feature pyramid achieves the efficient detection of small and large targets through feature fusion, and a new feature pyramid fusion mechanism is introduced to further improve detection performance. The more powerful object detection head part is optimized and improved, including an anchor frame clustering strategy, loss function design, and a category-balancing strategy, which allows YOLOv7 to achieve significant improvements in both detection accuracy and recall rate. In addition, YOLOv7 introduces various data enhancement techniques and training strategies, such as random cropping, scaling, flipping, and color transformation, as well as the dynamic adjustment of learning rate and progressive anchor frame matching, which effectively improve the generalization ability and performance of the model. In 2023, the YOLOv8 [27] model, which is the latest version in the YOLO object detection model and known for its joint detection and segmentation capabilities, was introduced. Featuring a novel architecture, enhanced convolutional layers in the backbone network, and a more advanced detection head, it stands as the optimal choice for real-time object detection. Additionally, YOLOv8 accommodates the latest computer vision algorithms, including instance segmentation, enabling the detection of multiple objects within an image or video. The model employs the Darknet-53 backbone network, known for its superior speed and accuracy compared to those of the preceding YOLOv7 network. YOLOv8 utilizes an anchorless detection head for predicting bounding boxes. The YOLOv8 model is more efficient than previous versions, improving accuracy and speed. YOLOv8 also incorporates a feature pyramid network for identifying objects of different sizes.

### 2.2. Model Lightness

Model lightweighting can be achieved by several technical means: compression, pruning, quantization, network structure design, and automated design [28,29]. Compression techniques can reduce the storage space of the model via parameter compression, weight sharing, low-rank decomposition, etc.; pruning techniques can reduce the computation and storage overhead of the model by removing unnecessary neurons, channels or layers, etc.; quantization techniques can reduce the storage space and computation overhead of the model by reducing the accuracy of the model; network structure design techniques can reduce the storage space and computation overhead of the model by designing lightweight network structures such as MobileNet [30] and ShuffleNet [31] to reduce the computational and storage overhead of the model; automated design techniques can be used to quickly search for lightweight network structures by automating the design of network structures, such as via network architecture search (NAS) [32]. Pruning is a common approach in model lightweighting, and its main idea is to reduce the computational and storage overhead of the model by removing unnecessary neurons, channels or layers, etc., so as to achieve lightweighting. Pruning can be applied to many mainstream deep learning models, including CNN, RNN, etc. The core of pruning is to determine which neurons, channels, or layers can be removed from a deep learning model. Common pruning methods include structural pruning [33] and nonstructural pruning [34]. Structural pruning refers to removing the entire structural units of the network, such as channels and layers. Structural pruning methods are commonly used in convolutional neural networks to reduce computation and storage overhead by removing unnecessary channels and layers. Nonstructural pruning refers to the removal of individual neurons or weights in the network and is commonly used in fully connected layers and recurrent neural networks. There are various pruning methods, including manual pruning and adaptive iterative pruning. Manual pruning requires manual pruning, which requires expertise and experience and is therefore less efficient. Threshold-based pruning is conducted by setting a threshold to filter out neurons, channels, or layers that have less impact on the model and removing them. Iterative pruning is used to gradually reduce the computational and storage overhead of the model via multiple pruning iterations [35]. Adaptive pruning is achieved by dynamically adjusting the pruning strategy, which is achieved by monitoring the operation of the model, and via the usage of computational resources. The mainstream methods of adaptive pruning are convolutional kernel pruning soft filter pruning (SFP) [35] and the traditional hard pruning method (hard filter pruning, HFP) [36].

### 2.3. Meter Reading

Meter reading is a technique that analyzes and processes meter data to extract useful information and that adjusts and optimizes the accordingly to actual needs. In the past few decades, scholars have studied and constructed several traditional methods to implement meter reading. These traditional methods are usually based on image processing and pattern recognition techniques such as image preprocessing, feature extraction, and classifier modeling steps. In image preprocessing, methods such as denoising, enhancement, and cropping are often used to reduce noise in images, increase the contrast of images, and crop irrelevant parts of images for the better analysis and processing of metered images. In terms of feature extraction, methods such as morphological operations, color features, and texture features are commonly used to extract useful information from epistatic images. Morphological operations can be used to detect edges and contours in metered images, color features can be used to detect color differences and variations in metered images, and texture features can be used to detect texture features and patterns in metered images. In terms of classifier modeling, methods such as artificial neural networks, support vector machines, and decision trees [6] are often used for the classification and recognition of tabulated images. The traditional methods mentioned above can learn and classify features in meter images for the purpose of meter literacy. Although the traditional methods have been widely used, they require manually designed features and have limited accuracy, especially in dealing with complex scenes and multiple types of meter meters, with low accuracy and efficiency. Deep-learning-based methods have been widely used in the field of meter reading. Deep-learning-based methods can learn the features of meters directly from the original meter images without manually designing features, and can better adapt to complex scenes and meet the needs of multi-type meters. For example, in electric power systems, researchers have used convolutional neural network-based methods for meter reading and achieved better results in experiments. Liu et al. [7] used the Faster R-CNN detection algorithm to locate meter locations, obtained high-quality images through the feature correspondence algorithm and perspective transformation techniques, and achieved meter reading by detecting pointer locations through Hough transform. In the water system, Zhang et al. [8] used deep learning methods for water meter reading and combined techniques such as multi-scale feature extraction and the attention mechanism to achieve better results. Therefore, traditional meter reading methods based on pattern recognition techniques require manual feature design and have limitations in terms of accuracy and efficiency. The deep learning-based meter reading method can learn features directly from the original meter image, and can adapt to the needs of complex scenes and multiple types of meters.

This paper proposes a YOLOv5-MRL model based on the original YOLOv5 model with lightweight improvements to address the limitations of traditional meter reading methods.

## 3. YOLOv5-MRL Network Structure

**Figure 1:**
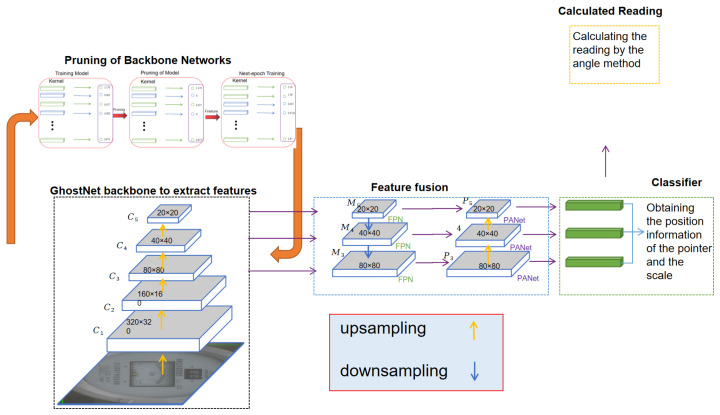
The proposed YOLOv5-MRL network structure.

The structure of the YOLOv5-MRL network is shown in Figure 1, which consists of four parts. These include the feature extraction part, where the feature extraction network adopts the cross stage partial (CSP) structure, which can obtain higher detection accuracy compared with the traditional ResNet due to its better computational and parametric efficiency. In the feature fusion part, the feature fusion layer is mainly used to fuse the features from different layers to improve the accuracy and robustness of object detection. In the classification and bounding box regression part, the detection head consists of several convolutional and fully connected layers, which are used to output the bounding box position and category information of each detected target. In the reading part, the angle method is used to calculate the readings of the pointer-type meter.

### 3.1. GhostNet-YOLOv5

YOLOv5 is an object detection model that can better meet the accuracy and speed requirements of real-time detection tasks. Although YOLOv5 shows good performance in the field of object detection, it is not suitable for deployment on embedded devices due to its large model size and large number of parameters. GhostNet requires fewer costs to generate more feature maps as a way to reduce the complexity of the network. Therefore, the introduction of GhostNet can effectively reduce the number of model parameters and computational complexity, thus improving the running speed and accuracy of the model, making it promising for object detection applications. In YOLOv5, GhostNet can better extract image features and thus improve the accuracy and efficiency of object detection. Therefore, in this paper, GhostNet-YOLOv5 is used, and a lightweight network mechanism is obtained by introducing GhostNet convolution into the YOLOv5 backbone network, CSPDarknet53, which has the advantages of YOLOv5 while compressing the YOLOv5 model’s size to a certain extent. Furthermore, it is used as the backbone network of the YOLOv5-MRL model in this paper.

**Figure 2:**
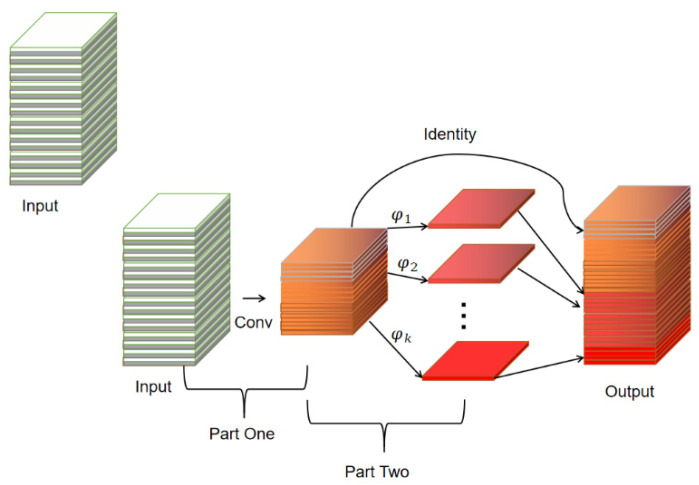
The Ghost module.

A Ghost convolution module is designed in GhostNet, which divides the normal convolution into two parts; the first part uses 1 × 1 convolution to obtain the necessary feature maps of the input features, and the second part performs layer-by-layer depth-separable convolution on the necessary feature maps obtained from the first part to obtain the Ghost feature maps. The Ghost module is shown in Figure 2.

In the conventional convolution operation, FLOPS denotes the computational operation complexity, which is calculated as shown in Equation (1):

(1) FLOPS=c×n×k×h′×w′

where c denotes the number of input channels, n denotes the number of output channels, k denotes the size of the convolution kernel, and h′ and w′ denote the output feature map size.

Assuming that one feature map has s-1 feature redundancy, the computational operation complexity generated by the Ghost module is divided into two parts; the first part is the complexity generated via ordinary convolution, $\frac{n}{s}\times h\prime \times w\prime \times c\times k\times k$, and the second part is the identity as well as the complexity generated by performing depth-separable convolution, $\left(s-1\right)\times \frac{n}{s}\times h\prime \times w\prime \times d\times d$. Then, the total complexity after using Ghost convolution is as shown in Equation (2):

(2) $$Ghost\_FLOPS=\frac{n}{s}\times h\prime \times w\prime \times c\times k\times k+\left(s-1\right)\times \frac{n}{s}\times h\prime \times w\prime \times d\times d$$

where c, n, k, h′, and w′ have the same definition as Equation (1). s represents the total mappings (1 intrinsic feature mapping and s-1 ghost special fold mapping) produced by each channel. n/s refers to the intrinsic feature mapping output obtained via ordinary convolution; d × d is the linear operation of average kernel size, which has a similar size to that of k × k.

The final FLOPS is reduced by a factor of s, as shown in Equation (3):

(3) $$s=\frac{c\times n\times k\times h\prime \times w\prime }{\frac{n}{s}\times h\prime \times w\prime \times c\times k\times k+\left(s-1\right)\times \frac{n}{s}\times h\prime \times w\prime \times d\times d}=\frac{c\times k\times k}{\frac{1}{s}\times c\times k\times k+\frac{s-1}{s}\times d\times d}\approx \frac{s\times c}{s+c-1}$$

From Equation (3), it can be seen that the computational operation complexity of ordinary convolution is s times that of GhostNet, which fully demonstrates the advantage of Ghost convolution over ordinary convolution in terms of computational volume. Therefore, Ghost-YOLOv5 is used as the backbone network of YOLOv5-MRL in this paper to effectively reduce the computational cost and compress the number of model parameters.

### 3.2. Soft Pruning of Convolution Kernels

**Figure 3:**
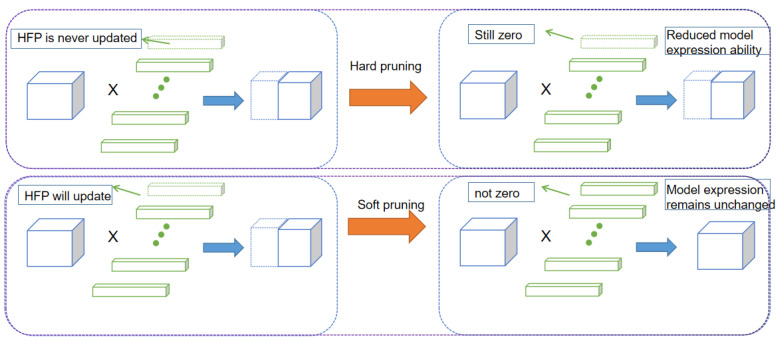
Soft pruning and hard pruning methods compared.

Convolutional kernel pruning SFP [35] is a method used to compress the parameters of deep neural networks and accelerate the inference process. The basic idea is to prune the unimportant convolutional kernels (i.e., redundant convolutional kernels) in the network model. The soft pruning method is more concise and effective than the traditional hard filter pruning (HFP) method [36]. HFP is the more popular pruning method. This method first ranks the convolutional kernels by metrics, and then sets a pruning rate for the layer to directly prune the kernels that do not meet the criteria. Next, the network model is fine-tuned according to the pre-training weights. During the fine-tuning process, the pruned convolutional kernels are directly discarded and do not participate in the subsequent iterative updates. However, the direct rounding of the convolutional kernels via this method leads to a larger decrease in the capacity of the network model, thus largely limiting the expressiveness of the network. In contrast, the major difference of SFP is that the clipped convolutional kernels still participate in the next iteration update. The method is able to retain more convolutional kernels to a large extent, thus maintaining the expressive capacity of the network model to a large extent while pruning the redundant convolutional kernels. SFP is effective in fine-tuning without using pre-training weights, because SFP prunes after each epoch, and after pruning, it performs further training and then continues pruning. Compared to HFP, SFP saves time in retraining, and is able to compress more parameters while maintaining the model’s effectiveness. This is because HFP is equivalent to dropping the convolution kernel directly, while 0 is not as extreme. The difference between the two pruning methods is shown in Figure 3.

**Figure 4:**
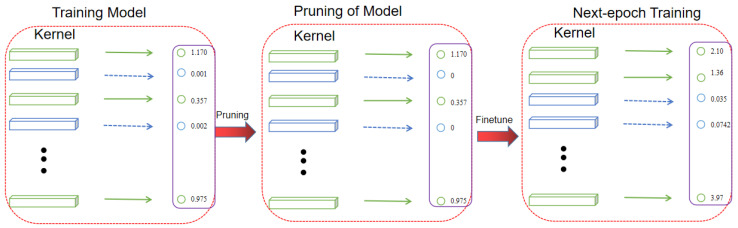
Convolution kernel pruning schematic.

When pruning is performed, each layer of the network model is first searched, and a suitable pruning rate is set for each layer. The pruning probability, $p_{i}$, and the number of convolutional kernels in layer i, $N_{i+1}$, are multiplied to represent the number of pruned convolutional kernels in a network layer, and the $N_{i+1}\times p_{i}$ kernels with the lowest $L_{2}$ parametric number of convolutional kernels are pruned, which is achieved by setting the value of that convolutional kernel to 0, thus completing the pruning operation of the i-th layer of convolutional kernels. Thus, with the iteration of epoch, the final model will contain some convolutional kernels with a value of 0, and these kernels will be removed directly to obtain the final model. The pruning schematic is shown in Figure 4.

Suppose the number of input feature channels in layer i is $N_{i}$, the number of output channel features is $N_{i+1}$, the dimension of the input feature map is $H_{i}\times W_{i}$, and the dimension of the output feature map is $H_{i+1}\times W_{i+1}$; the input feature dimension in layer i will be $N_{i}\times H_{i}\times W_{i}$, and the output feature dimension will be $N_{i+1}\times H_{i+1}\times W_{i+1}$. Let the pruning rate of layer i be $P_{i}$; when pruning, $N_{i+1}\times P_{i}$ convolution kernels need to be set to zero, only $N_{i+1}\times (1-P_{i})$ convolution kernels are valid, and the output feature dimension of layer i after pruning becomes $N_{i+1}\times \left(1-P_{i}\right)\times H_{i+1}\times W_{i+1}$. Pruning is continued for layer i + 1, and the pruning rate is expressed by $P_{i+1}$. The computation volume of layer i + 1 before pruning is ${N_{i+2}\times N}_{i+1}\times k^{2}\times H_{i+1}\times W_{i+1}$. The computation after pruning becomes ${N_{i+2}\times \left(1-P_{i+1}\right)\times N}_{i+1}\times \left(1-P_{i}\right)\times k^{2}\times H_{i+1}\times W_{i+1}$. In the above equation, $N_{i+2}\times H_{i+2}\times W_{i+2}$ denotes the output feature map size of layer i + 1. Each point on the feature map needs to go through $N_{i+1}\times k^{2}$ computational quantities to be obtained. After pruning, the number of input feature channels becomes $N_{i+1}\times \left(1-P_{i}\right)$ and the number of output channels becomes $N_{i+2}\times \left(1-P_{i+1}\right)$ mainly after the change of output channels and input channels. The multiplier of the calculated quantity before and after pruning can be obtained via division, as shown in Equation (4):

(4) $$\left(1-P_{i+1}\right)\left(1-P_{i}\right)=\frac{{N_{i+2}\times \left(1-P_{i+1}\right)\times N}_{i+1}\times \left(1-P_{i}\right)\times k^{2}\times H_{i+1}\times W_{i+1}}{{N_{i+2}\times N}_{i+1}\times k^{2}\times H_{i+1}\times W_{i+1}}$$

The final reduced computation is obtained by subtracting that multiple from 1. The final reduced computation is as follows:

(5) $$1-\left(1-P_{i+1}\right)\left(1-P_{i}\right)$$

Therefore, this paper uses a soft pruning algorithm of the convolutional kernel to prune the YOLOv5-MRL backbone network to remove redundant parameters, thus making the model more efficient. At the same time, pruning can also make the model more robust and reduce the risk of overfitting. This is because pruning can make the model more stable and avoid overfitting the training data by removing excessive parameters.

### 3.3. Dial Number Recognition and Reading

**Figure 5:**
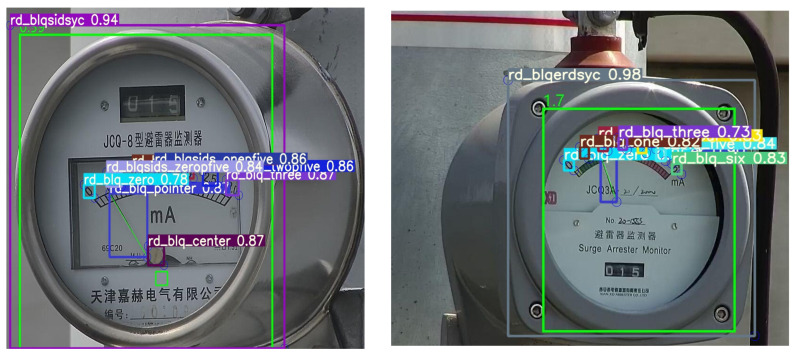
YOLOv5-MRL recognition effect, from left to right are the JCQ-8 lightning rod monitor and JCQ3A lightning rod detector.

Zou et al. [37] propose a new automatic meter reading method that can accurately fit elliptical and circular dials using the method of fitting a circle, which requires the coordinate values of the dial scale value, the pointer, the center point of the dial, and the six scale targets to be calculated. The trained model is first used to locate the corresponding candidate region from the original image, and then the outer circle of the dial is fitted using the above parameters, as shown in Figure 5 below.

The specific steps of the method [37] are as follows: (1) Instrument detection: use the model to detect the positions of the instrument panel, pointers, and tick marks, and obtain the center coordinates of the circle by fitting the minimum outer circle. In order to avoid errors, it is necessary to use the minimum outer circle of the ellipse. (2) Calculation of reading: the scale value indicated by the pointer is determined by calculating the angle between the pointer and each scale. First, the angle between the pointer and the 0 scale is calculated, and the angles of the other scales are calculated on this basis. The scale value indicated by the pointer is determined based on the minimum positive and maximum negative angles. (3) Output of results: the results of the recognition are output, including the scale value indicated by the pointer and the recognized image. The method has the following advantages: Firstly, it can be applied to various types of meters. Secondly, it enables fast and accurate readings. Therefore, this dial fitting algorithm is also used in this paper for meter reading.

## 4. Experimental and Results Analysis

### 4.1. Experimental Environment

In this paper, the hardware configuration used for the experiments is Intel Core i7-9700K processor and NVIDIA RTX3090 card, and the software environment is CUDA10.0 and the PyTorch3.6 deep learning framework. All models were trained, validated, and tested under the same hyperparameters. The intersection over union (IoU) between the actual target and the monitored target [38] was used in all detections to evaluate whether or not the location of the target was successfully predicted, and a predicted target with IoU > 0.5 was considered to be the location of the target successfully predicted. Evaluating the performance of the network model is required to take into account both accuracy and recall (Precision), and recall (Recall), and mean average precision (mAP) is generally used in object detection to evaluate the performance of the network model. Equation (6) is the formula for accuracy, and Equation (7) is the formula for recall. The specific parameters are shown in Table 1 below.

(6) $$Precision=\frac{TP}{TP+FP}$$

(7) $$Recall=\frac{TP}{TP+FN}$$

The average precision (AP) is defined as the average of the precision rates under different recall rates. mAP is the average of the detection precision of all target categories, and is generally used to evaluate the overall performance of the network. The mAP calculation formula is shown in Equation (8):

(8) $$mAP=\frac{\sum_{i=1}^{n}AP_{i}}{n}$$

where APi is the detection accuracy of a category, and n is the number of categories.

### 4.2. Lighting-Meter Experimental Dataset

In this paper, a new dataset called Lighting-Meter is constructed, which contains 2000 lightning rod images and 32,000 labeled targets. When collecting the substation meter dataset, it is difficult to generate the complete ideal dataset due to the high acquisition cost. Moreover, the number of training samples for different categories of meters varies greatly, leading to data imbalance. Therefore, we use data augmentation to extend the size and diversity of certain types of data via strategies such as scaling, flipping, panning, cropping, and color brightness transformation to improve the robustness and generalization of detection models. The data enhancement strategy solves the problem of lack of texture features of metered appearance to some extent.

**Figure 6:**
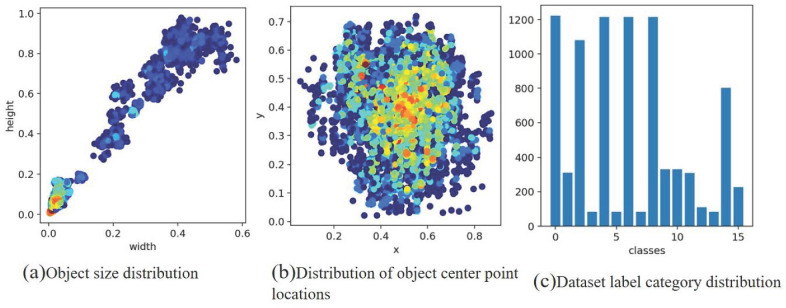
The result of k-means clustering with the cluster center set to 16.

The dataset containing four types of images are the JCQ-8 lightning rod detector, JCQ-8H lightning rod detector, JCQ3A lightning rod monitor, and JCQ3A-20/2000 lightning rod monitor, and the number of each of the two types is 500. Labeling involves using the VOC2007 format, labeling the position of the pointer (rd_pointer) and the center of the pointer (rd_center), at a scale of zero (rd_zero), a scale of zero point five (rd_zeropfive), a scale of one (rd_one), a scale of one point five (rd_onepfive), a scale two (rd_two), a scale of two point five (rd_twopfive), a scale of three (rd_three), a scale of four (rd_four), a scale of five (rd_five), and a scale of six (rd_six). The JCQ3A-20/2000 lightning rod (rd_blqerdsyc), JCQ-8H-type lightning rod (rd_blqsan_bp), JCQ-8-type lightning rod (rd_blqsidsyc), and JCQ3A lightning rod (rd_blqyidsyc) are labeled to keep the center of the labeled box on the scale as much as possible. The ratio of the randomly selected training set to the test set is 7:3, i.e., there are 1400 images in the training set and 600 images in the test set. The images were recorded using the xml format with all the annotated positions and category information. To enhance the prior understanding of the dial target, this study applies the K-means algorithm to produce 16 prior frames using the Lighting-Meter dataset, and the resulting clusters are depicted in Figure 6. Figure 6a illustrates the clustering of target sizes, while Figure 6b illustrates the clustering of target centroid locations, and Figure 6c presents the statistics of different label types within the dataset.

### 4.3. YOLOv5-MRL Literate Model Effect

**Figure 7:**
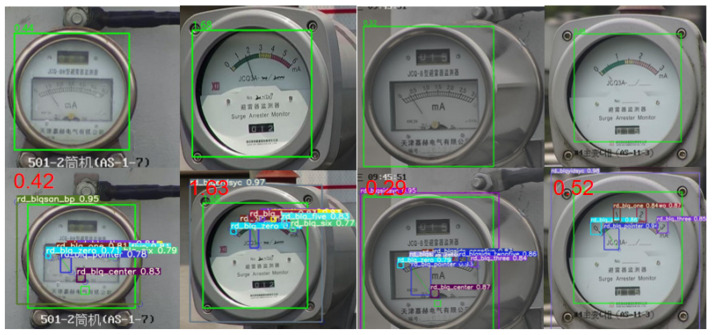
YOLOv5-MRL recognition effect. The first and third columns of the above diagram show the JCQ-8 lightning rod monitor and the second and fourth columns show the JCQ3A lightning rod detector. The first row is the original picture and the second row is the schematic of the test result. Where rd_blq_zero means scale 0, rd_blq_one means scale 1, rd_blq_onepfive means scale 1.5, rd_blq_two means scale 2, rd_blq_twopfive means scale 2.5, rd_blq_three means scale 3, rd_blq_four indicates scale 4; rd_blq_s, rd_blq_five means scale 5, rd_blq_six means scale 6.

In this paper, a comparison experiment is used to verify the validity of the YOLOv5-MRL model, using the mean error, δ, as an evaluation index, as shown in Equation (9):

(9) $$\delta =\frac{\sum_{i=1}^{n}\left|a_{i}-A_{i}\right|}{n}$$

n denotes how many sets of experimental data there are, $a_{i}$ denotes the scale predicted by the model, and $A_{i}$ denotes the number of manual readings. In this paper, 10 sets of data were used, and the literacy effect is shown in Table 2:

As can be seen from the data in Table 2, in terms of inference time and number of parameters, the number of parameters of the YOLOv5-MRL model proposed in this paper is only 3.8 MB, which is 47.2% lower compared with that of the original YOLOv5s model. Compared with that of YOLOv3 and YOLOv3-spp, it is reduced by 37.7% and 38.7%, respectively, and compared with that of Tiny-YOLOv7 and YOLOv8s, it is reduced by 37.7% and 47.2%, respectively. In terms of inference time, YOLOv5-MRL is small and fast, with an inference time of 5.0 ms/frame, meeting the real-time detection requirements. It is more suitable for applications in micro and small embedded devices. In terms of average error rate, that of YOLOv5-MRL is slightly higher than that of the original YOLOv5s model as well as that of the YOLOv3 and YOLOv3-spp models, but the recognition effect is within a 0.05 error, as shown in Figure 7, which means it can better achieve the detection of meter scale.

Therefore, since YOLOv5-MRL uses a more lightweight backbone network and prunes the backbone network using a soft pruning algorithm with a convolutional kernel, YOLOv5-MRL’s prediction results compared with those of the original YOLOv5s object detection model can maintain good accuracy, and it can also have a faster detection speed.

### 4.4. Experimental Analysis of Different Pruning Rates

**Figure 8:**
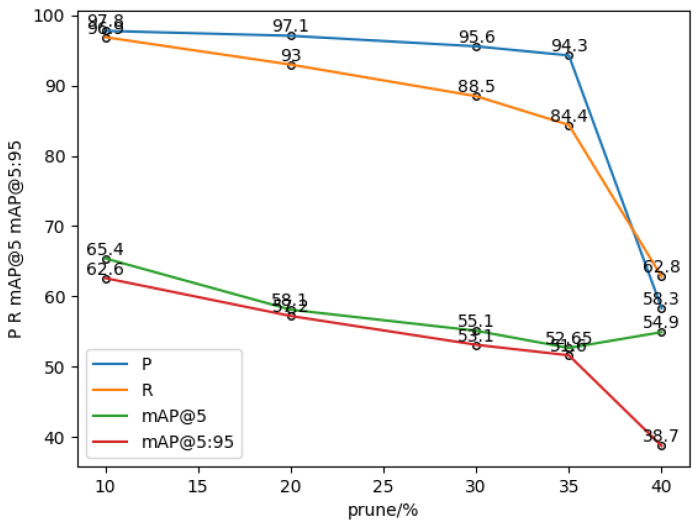
Graph of test results for different pruning rates.

In order to obtain the optimal pruning rate, as shown in Table 3, this paper adopts the soft pruning strategy of convolutional kernels to soft-prune YOLOv5. Firstly, the weights of different convolutional kernels are calculated by using L_2 regularization, then different network pruning rates are set to prune the network structure, models of different sizes are obtained, and finally the pruned models are retrained and tested on the Lighting-Meter dataset. The pruning rates in the experiments were set to 10%, 20%, 30%, 35%, and 40%, and the model’s performance after pruning at different rates is shown in Figure 8. In Table 3, Prune10% denotes the model with a pruning rate of 10%; P denotes Precision, R denotes Recall (recall), mAP@5 represents the mean average precision at an intersection over union (IoU) threshold of 0.5. We calculate the average value of the AP for each category. The statement means that mAP@5 is the average precision calculated at an IoU threshold of 0.5. We calculate the average value of the AP for each category, and mAP@5:95 means that we can calculate the average of the AP of each category when IoU is set to (0.5~0.95).

### 4.5. Ablation Experiments

In this section, ablation experiments are conducted to evaluate the impact of the ghost backbone network and soft pruning methods on the performance of the object detection algorithm under the same experimental conditions. The experimental results are shown in Table 4. The YOLOv5s model from Ultralytics version 5.0 was chosen as the baseline model for the ablation experiments. Let the resolution size of the input image be 640 × 640; the experimental results of training 300 epochs are shown in Table 3. The data in the table show that the introduction of YOLOv5s into the ghost backbone network caused a certain loss of accuracy, but the detection speed of the model increased many times, allowing the model to be effectively compressed the detection speed of the network to be improved. after YOLOv5s used SFP (soft pruning of channels), the accuracy of the model remained almost the same, the size of the model was reduced by 50%, and the detection speed increased a lot. This shows that the soft pruning of convolutional kernels used in this paper can effectively remove the redundant parameters from the model, making it more efficient.

### 4.6. Comparison Experiment

In order to verify in detail the detection of meter scales by YOLOv5-MRL, this section validates the YOLOv5-MRL model by comparing it with a variety of state-of-the-art object detection models. The experiment was conducted by comparing the YOLOv5-MRL model with Ultralytics 5.0 versions; YOLOv3 [39], YOLOv3-spp, Tiny YOLOv3, and Tiny YOLOv4 [40], YOLOv5 [24], YOLO-Nano [14], Tiny-YOLOv7 [26], and YOLOv8s [27] models were compared, and the models were all trained and validated using the Lighting-Meter dataset.

**Figure 9:**
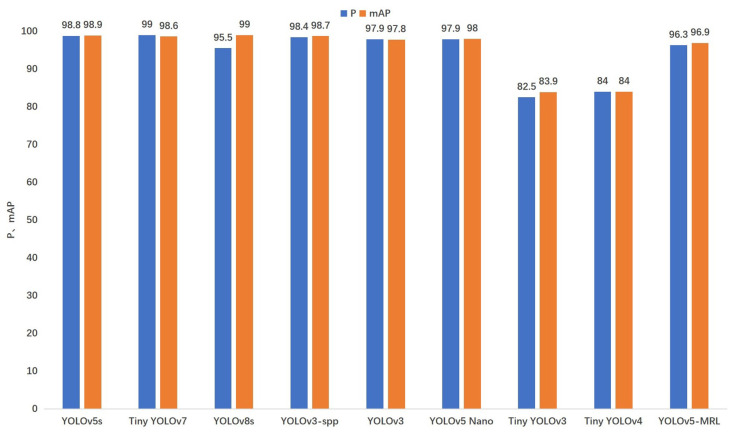
Comparison of different object detection models.

As shown in Table 5 and Figure 9, this section focuses on weight size (Weights), Precision, mean accuracy precision (mAP), and speed of computation (Inference) comparisons. As can be seen from Table 5, in terms of the model’s size and number of parameters, the size of the YOLOv5-MRL model proposed in this paper is only 5.5 MB, which is 98.48% lower compared to that of the original YOLOv5s model. Compared with that of YOLOv3 and YOLOv3-spp, it is reduced by 98.82% and 98.84%, respectively. In terms of the number of model parameters, the reduction is 36.6% compared to that in Tiny YOLOv4. Compared to the latest Tiny-YOLOv7 and YOLOv8s models, YOLOv5-MRL is smaller and faster, making it more suitable for use in micro and small embedded devices, while the YOLOv5-MRL detection speed of 5.0 ms/frame meets the real-time detection requirements. As shown in Figure 9, the mean accuracy precision of YOLOv5-MRL is 13.0% higher than that of Tiny YOLOv3 and 12.9% higher than that of Tiny YOLOv4 in terms of model accuracy. Compared to the original YOLOv5s model as well as YOLOv3 and YOLOv3-spp, YOLOv5-MRL is still able to maintain an accuracy of 96% in terms of mean accuracy precision, and is able to achieve the better detection of meter scales, although there is a drop in mean accuracy precision. Compared to the lightweight YOLO-Nano model, the mean accuracy precision of the YOLOv5-MRL model is comparable to that of the YOLO Nano model, but that of the model YOLOv5-MRL is smaller. As can be seen from Table 5, in terms of runtime, YOLOv5-MRL is able to achieve faster detection compared to models such as YOLOv5s and YOLOv3-spp, as well as the latest Tiny-YOLOv7, YOLOv8s, etc., requiring an average processing time of only 5 ms per image. This is due to the use of a lighter backbone feature extraction network and a soft pruning strategy of the convolutional kernel on the backbone network to remove redundant parameters, improving operational efficiency while maintaining detection accuracy. Deep learning models with slower inference speeds may take longer to complete the inference task, leading to an increase in the energy consumption of the system, as well as an increase in the burden on the environment. Therefore, fast reasoning speeds can help reduce energy consumption and minimize the impact on the environment. Therefore, the YOLOv5-MRL model is well suited for ground-up deployment because of its extremely fast inference speed and minimal energy consumption.

## 5. Conclusions

In this paper, we propose a lightweight object detection model, YOLOv5-MRL, which aims to address the challenges of deploying traditional object detection models on edge devices and ensure the detection accuracy and real-time performance of the lightweight model. The backbone feature extraction network of the YOLOv5-MRL model adopts a lighter-weight Ghost network, and uses the convolutional kernel soft pruning algorithm to prune the backbone feature extraction network. The soft pruning algorithm is used to prune the backbone feature extraction network and complete the compression of the network structure. The algorithm effectively compresses the model size, eliminates redundant parameters, and realizes the better recognition of pointer meters while ensuring high detection accuracy and real-time performance.

However, the YOLOv5-MRL algorithm brings more parameters to the model by predetermining the size of the a priori frame in the Anchor parameters, affecting the detection speed. In the future, considering the algorithm still has room for improvement, we will optimize the algorithm with the latest YOLOv8 algorithm because the current model is only for a single scene, so we need to add more data to improve the generalization ability of the proposed model to create a generalized meter reading model. At the same time, we will also enhance the interpretability of the algorithm in order to improve the robustness of the meter reading model.

## Figures and Tables

**Table 1:** Parameter settings.

Parameter Names	Parameter Values
Weight_decay	0.0005
Momentum	0.937
Learning_rate	0.01
Batch_size	4
Epochs	600

**Table 2:** Comparison of YOLOv5-MRL and other object detection models.

Model	Average Error	Inference/ms	Para/MB
YOLOv3	0.027	27.5	6.1
YOLOv3-SPP	0.025	28.0	6.2
YOLOv3-Tiny	0.036	6.0	0.8
YOLOv4-Tiny	0.045	3.6	6.0
YOLOv5s	0.020	29.0	7.2
YOLO-Nano	0.01	3.6	1.9
Tiny-YOLOv7	0.024	7.8	6.1
YOLOv8s	0.022	40.6	7.2
YOLOv5-MRL	0.029	5.0	3.8

**Table 3:** Test results for different pruning rates.

Pruning Rate	Precision	Recall	mAP@5	mAP@5:95
Prune10%	97.8	96.9	65.4	62.4
Prune20%	97.1	93	58.1	57.2
Prune30%	95.6	88.5	55.1	53.1
Prune35%	94.3	84.4	52.65	51.6
Prune40%	58.3	62.8	54.9	38.7

**Table 4:** Ablation experiments.

Model	Weights/MB	Precision/%	Recall/%	mAP@:5(%)	mAP@5:95(%)	Inference/ms
YOLOv5s	362	79.4	98.6	98.6	71.0	424
YOLOv5s + ghost	21.6	74.9	98.0	97.6	67.3	6.2
YOLOv5s + SFP	181	81.7	98.4	98.3	66.6	27.6

**Table 5:** Comparison of the results of different detection algorithms.

Model	Weights/MB	Para/MB	Inference/ms
YOLOv5s	362	7.2	29
YOLOv3-spp	478	6.2	28
YOLOv3	470	6.1	27.5
Tiny YOLOv7	71.4	6.1	7.8
YOLOv8s	21.4	7.2	40.6
YOLO-Nano	14.3	1.9	3.6
Tiny YOLOv3	66.48	0.8	6.0
Tiny YOLOv4	23.7	6.0	3.6
YOLOv5-MRL	5.5	3.8	5.0

## Data Availability

Restrictions apply to the datasets.
